# Dietary substitution with space-mutated *Arundo donax* L. remodels carnitine-mediated lipid oxidation and improves mutton flavor in Hu sheep

**DOI:** 10.1016/j.fochx.2026.104555

**Published:** 2026-10-06

**Authors:** Yajing Nie, Liming Zhang, Chengxing Han, Xujie Li, Yi Wu, Chunhui Wang, Youji Ma

**Affiliations:** a College of Animal Science and Technology, Gansu Agricultural University, Lanzhou 730070, China; b Gansu Key Laboratory of Animal Generational Physiology and Reproductive Regulation, Lanzhou 730070, China

**Keywords:** Hu sheep, Acylcarnitine, Long-chain fatty acids, Oxidation, Volatile compounds

## Abstract

Space-mutated *Arundo donax* L. (SA) is a novel stress-tolerant forage crop developed through space-induced mutagenesis. The study aimed to investigate how SA modulates lamb flavor through carnitine-mediated lipid metabolic remodeling and alterations in volatile flavor profiles. In this study, thirty Hu lambs were randomly assigned to three dietary groups: control (CK), SA replacing alfalfa (T1), and SA replacing protein supplements (T2). As a result, lipidomics identified 1098 lipids, including 113 and 98 differential lipids in CK vs. T1 and CK vs. T2, respectively. HS-SPME-GC–MS detected 535 volatile compounds, with 201 and 30 differential volatile compounds, respectively. Relative odor activity value (ROAV) analysis identified 43 key odor-active compounds (ROAV ≥1). Correlation analysis revealed significant positive associations between acylcarnitines and key volatile compounds. These findings provide new insights into SA-mediated lipid metabolic remodeling and flavor regulation, supporting its potential as a nutritional strategy for improving lamb flavor.

## Highlights

• Integrated lipidomics and flavoromics deciphered the flavor genesis in sheep fed space-mutated *Arundo donax*.
• Space-mutated *Arundo donax* feeding improved meat tenderness by reducing muscle fiber diameter and cross-sectional area.
• Downregulation of long-chain acyl carnitines indicated a suppression of the mitochondrial carnitine shuttle.
• Attenuated oxidative flux mitigated off-flavors and promoted fruity ester accumulation.

## 1 Introduction

Mutton is a nutrient-dense meat rich in high-quality protein, vitamins, and essential fatty acids, making it well suited to the growing consumer demand for nutritious and health-oriented meat products (Li et al., 2022; Rodrigues et al., 2023). However, its characteristic flavor may limit broader consumer acceptance. This distinctive flavor is closely associated with lipid-derived compounds, particularly branched-chain fatty acids such as 4-methyloctanoic and 4-methylnonanoic acids, as well as specific aldehydes, ketones, and phenolic compounds (Wu et al., 2023). As an important reservoir of lipid-soluble flavor precursors, adipose tissue plays a key role in shaping the sensory characteristics of mutton through lipid deposition and metabolism (Calkins & Hodgen, 2007). During cooking, fatty acids and other flavor precursors undergo thermal degradation and oxidation, generating a diverse range of volatile compounds, including aldehydes, ketones, alcohols, hydrocarbons, and lactones, which collectively contribute to the aroma and flavor of cooked mutton (Bleicher, Ebner, & Bak, 2022; Fu, Cao, Yang, & Li, 2022). Consequently, elucidating the metabolic regulation of lipid composition is important for understanding and modulating flavor formation and, ultimately, enhancing meat quality.

The intensification of animal husbandry has increased the demand for high-quality feed resources, highlighting the need to develop alternative protein-rich forages. Giant reed (*Arundo donax* L.) is a perennial grass characterized by high biomass production and broad environmental adaptability. However, its use as forage is limited by its relatively high fiber content and poor overwintering performance (Nsanganwimana, Marchand, Douay, & Mench, 2014). Space-induced mutagenesis offers a promising approach for generating novel plant germplasm with improved agronomic and nutritional traits (Jia et al., 2024; Maffei et al., 2024). In 2013, buds of *Arundo donax* L. were exposed to the space environment aboard the Shenzhou-10 spacecraft and subsequently propagated by tissue culture and subjected to successive rounds of ground-based selection, ultimately yielding space-mutated *Arundo donax* L. (SA). Previous studies have demonstrated that space-induced mutagenesis can generate extensive phenotypic variation, enabling the selection of mutant lines with improved growth, biomass-related traits, and stress tolerance (Kuang, He, Huang, & Jiang, 2025). SA retains the stress tolerance of its parental material while exhibiting improved nutritional characteristics. Our preliminary analysis showed that, on a dry-matter basis, SA hay contained 19.12% crude protein (CP), 1.15% ether extract (EE), 65.39% neutral detergent fiber (NDF), and 41.06% acid detergent fiber (ADF). Feeding trials further demonstrated that SA could be incorporated into Hu sheep diets without adversely affecting normal growth and development, supporting its potential as a novel forage resource.

Forage composition and feeding strategies can influence meat quality and flavor by modulating muscle lipid and amino acid metabolism (Li et al., 2026; Sun, Wang, Kuang, Fan, & Yang, 2025; Zhang et al., 2025). Among the various flavor precursors, fatty acids are particularly important because they serve not only as major metabolic substrates but also as key precursors of lipid-derived volatile flavor compounds. Mitochondrial fatty acid oxidation is tightly regulated by the carnitine shuttle, which mediates the transport of long-chain fatty acids (LCFAs) into the mitochondrial matrix. Carnitine palmitoyltransferase 1 (CPT1), located on the outer mitochondrial membrane, catalyzes the conversion of long-chain fatty acyl-CoA to acylcarnitine and represents a key regulatory step in carnitine-dependent mitochondrial LCFA transport and subsequent β-oxidation (Liang, 2023; Smith, Choi, & Wolfgang, 2025). Increasing evidence suggests that plant-derived secondary metabolites and other forage bioactive compounds can modulate antioxidant capacity and lipid metabolism in livestock (Diao, Zhang, Hao, Mu, & Zhang, 2025; Guo et al., 2025; Yu et al., 2026). However, the potential role of forage-derived bioactive metabolites in regulating the carnitine shuttle in ruminant skeletal muscle remains poorly understood. In particular, it is unclear whether these metabolites influence carnitine-dependent mitochondrial transport of LCFAs and whether such changes are associated with alterations in intramuscular lipid composition and the formation of lipid-derived volatile flavor compounds. Based on this rationale, we hypothesized that secondary metabolites derived from SA may modulate carnitine-related fatty acid metabolism in ruminant skeletal muscle, thereby influencing the mitochondrial transport and utilization of LCFAs and contributing to muscle lipidome remodeling and associated changes in the volatile flavor profile.

To investigate this hypothesis, LC-MS/MS-based lipidomics and HS-SPME-GC–MS-based flavoromics were integrated to systematically characterize changes in muscle lipid composition and volatile profiles in Hu sheep fed SA, with particular emphasis on lipid metabolic features associated with the carnitine shuttle and fatty acid metabolism. A lipid-volatile correlation network was further constructed to explore potential links between carnitine-related lipid remodeling and flavor formation. This integrated approach provides new evidence for a potential carnitine shuttle-lipid remodeling-flavor axis associated with the effects of SA feeding on mutton quality.

## 2 Materials and methods

### 2.1 Chemicals and reagents

All solvents and chemicals used were of HPLC or LC-MS grade. Acetonitrile (ACN), methanol (MeOH), isopropanol (IPA), dichloromethane (CH_2_Cl_2_), methyl tert-butyl ether (MTBE), and n-hexane were obtained from Merck (Darmstadt, Germany). Formic acid (FA) and ammonium formate (AmFA) were obtained from Sigma-Aldrich (St. Louis, MO, USA). Analytical grade sodium chloride (NaCl) was supplied by Sinopharm Chemical Reagent Co., Ltd. (Shanghai, China). Ultrapure water was generated using a Milli-Q water purification system (Millipore, Billerica, MA, USA). Lipid standards for lipid identification were obtained from Sigma-Aldrich (St. Louis, MO, USA) and Avanti Polar Lipids (Alabaster, AL, USA). The SPLASH® LIPIDOMIX® Mass Spec Standard (Avanti Polar Lipids, Alabaster, AL, USA; Cat. No. 330707) was used as the internal standard mixture for lipidomic analysis. Detailed information on the lipid internal standards used for LC-MS/MS analysis is provided in Table S1.

### 2.2 Animals and samples collection

This experiment selected 30 Hu lambs (3-month-old; initial body weight: 20.95 ± 1.09 kg) with homogeneous genetic backgrounds and physiological conditions were used as experimental animals. The lambs were sourced from the Xinyuan Modern Agricultural Science and Technology Demonstration Park (Honggu District, Lanzhou City, Gansu Province, China) and randomly divided into three groups (10 lambs/group): control group (CK), replacing alfalfa with SA group (T1), and replacing protein supplements (Soybean meal and Cotton meal) in substitute concentrates with SA group (T2), The basal ration was developed in compliance with the national standard “Nutrient Requirements of Meat-Type Sheep and Goats” (NY/T 816-2021), with detailed compositions and nutritional levels provided in Table 1. The experiment lasted a total of 67 days, which included a pre-experimental phase of 7 days and a formal testing period of 60 days. The lambs were fed twice daily at 09:00 and 18:00 following standard farm protocols, with unrestricted access to drinking water throughout the study. The pens were maintained in a clean, dry, and periodically sanitized condition.

**Table 1:** Composition and nutritional levels of the basal diet (dry matter basis).

Ingredient (%)	Content (%)		
	CK	T1	T2
Ground corn	35.75	35.75	35.75
Wheat bran	10	10	10
Alfalfa hay	30	0	30
Wheat straw	10	10	10
Space-mutated *Arundo donax* L*.*	0	30	12.86
Soybean meal	7.28	7.28	0
Cotton meal	5.58	5.58	0
NaCl	0.25	0.25	0.25
Premix	1.14	1.14	1.14
Total	100	100	100
Nutritional levels (%)			
CP	16.36	17.32	15.69
EE	2.39	2.38	2.49
NDF	30.32	33.12	32.90
ADF	18.01	20.52	20.17
Ca	1.27	1.17	1.52
P	0.55	0.53	0.47
ME/(MJ/kg)^2^	10.80	10.49	11.02

Note: CP, crude protein; EE, Ether Extract; NDF, neutral detergent fiber; ADF, acid detergent fiber. The premix provided per kg of diet: Fe (as ferrous sulfate) 45 mg, Cu (as copper sulfate) 14 mg, Zn (as zinc sulfate) 40 mg, Se (as sodium selenite) 0.25 mg, I (as potassium iodide) 0.25 mg, Co 0.30 mg, Mn (as manganese sulfate) 40 mg, VA 3000 IU, VD 500 IU, VE 20 IU. ME was a calculated value, according to *Nutrient Requirements for Meat Sheep* (NY/T816-2021), while the others were measured values.

Following the feeding trial, the humane slaughter of lambs on the farm is carried out by qualified professionals under the supervision of veterinarians in compliance with animal welfare standards. Lambs subjected to a 12 h fasting period, with free access to drinking water throughout the process. Randomly selected 6 experimental lambs of similar weight from each group for slaughter and sample collection (*n* = 6). The lambs were slaughtered using electrical stunning followed by exsanguination. Samples of the left longissimus thoracis muscle were collected within 30 min postmortem, immediately transferred into cryovials, and snap-frozen in liquid nitrogen. Then samples were transported to the laboratory in liquid nitrogen within 2 h and subsequently stored at −80 °C until further analysis.

### 2.3 Muscle histomorphology and intramuscular fat (IMF) content

For histomorphological analysis, fixed muscle samples were trimmed into tissue blocks (approximately 1 cm × 2 cm × 2 cm), dehydrated through a graded ethanol series, embedded in paraffin, and sectioned at a thickness of 4 μm. The sections were stained with hematoxylin and eosin (H&E) following standard histological procedures. Digital slides were acquired using a Panoramic DESK scanner (3DHISTECH Ltd., Budapest, Hungary). And morphometric analysis was subsequently performed using Case Viewer 2.3 software. For each group, four non-serial sections were evaluated. And at least five random fields of view per section were captured at 100 × magnification. Myofiber diameter, cross-sectional area, and density were quantified, and the mean values were used for subsequent analysis. IMF content in muscle samples was determined using the Soxhlet extraction method according to the National Food Safety Standard of China (GB 5009.6-2016).

### 2.4 Determination of CPT1 and oxidative stress markers

The concentrations of CPT1, malondialdehyde (MDA), and reactive oxygen species (ROS) in the *longissimus dorsi* muscle samples were determined using commercial enzyme-linked immunosorbent assay (ELISA) kits (CPT1, JX-60303B2; MDA, JX-9321B2; ROS, JX-60302B2; Yancheng Junxing Biotechnology Co., Ltd., China). All assays were performed according to the manufacturer's instructions.

### 2.5 HPLC-MS/MS analysis

Samples were thoroughly ground in liquid nitrogen, and 20 mg of each sample was accurately weighed and transferred into a microcentrifuge tube. Subsequently, 1 mL of extraction solvent (MTBE:MeOH = 3:1, v/v) containing the internal standard mixture was added. The samples were vortexed for 15 min, followed by the addition of 200 μL of ultrapure water. After further vortexing for 1 min, the samples were centrifuged at 12,000 rpm for 10 min. A 200 μL aliquot of the upper organic phase was collected and evaporated to dryness using a vacuum concentrator. The dried extracts were reconstituted in 200 μL of ACN:IPA (1:1, v/v) and subjected to LC-MS/MS analysis.

Lipidomic analysis was performed using an LC-ESI-MS/MS system consisting of an ExionLC™ AD UPLC system coupled with a QTRAP® 6500+ mass spectrometer (AB SCIEX, Framingham, MA, USA). Chromatographic separation was achieved using a Thermo Accucore™C30 (2.6 μm, 2.1 mm × 100 mm i.d.). The mobile phases consisted of solvent A (acetonitrile/water = 60/40, v/v, containing 0.1% formic acid and 10 mmol/L ammonium formate) and solvent B (acetonitrile/isopropanol = 10/90 v/v, containing 0.1% formic acid and 10 mmol/L ammonium formate). The gradient elution program was set as follows: A/B = 80:20, v/v at 0 min, 70:30 v/v at 2 min, 40:60 v/v at 4 min, 15:85 v/v at 9 min, 10:90 v/v at 14 min, 5:95 v/v at 15.5 min, 5:95 v/v at 17.3 min, 80:20 v/v at 17.3 min, 80:20 v/v at 20 min. The flow rate was 0.35 mL/min, the column temperature was maintained at 45 °C, and the injection volume was 2 μL. Mass spectrometric analysis was performed using an electrospray ionization (ESI) source operated in both positive and negative ion modes. The source temperature was set at 500 °C, with ion spray voltages of 5500 V in positive and −4500 V in negative modes, respectively. The ion source gases GS1, GS2, and curtain gas (CUR) were set at 45, 55, and 35 psi, respectively. Instrument tuning and mass calibration were performed using polypropylene glycol solutions at concentrations of 10 and 100 μmol/L in triple quadrupole (QQQ) and linear ion trap (LIT) modes, respectively. QQQ scans were acquired in multiple reaction monitoring (MRM) mode using nitrogen as the collision gas (5 psi). Optimized declustering potential (DP) and collision energy (CE) values were applied for individual MRM transitions. Quality control (QC) samples were prepared by mixing equal volumes of all experimental samples. QC samples were injected after every 10 samples to monitor analytical stability and reproducibility. Lipid species were annotated using a scheduled multiple reaction monitoring (sMRM)-based strategy. Identification was performed based on precursor ion (Q1), characteristic product ion (Q3), retention time (RT), and lipid-class-specific diagnostic fragment ions, with reference to the self-established MWDB lipid database (http://www.metware.cn/). For sphingolipid species, including ceramide (Cer) and Hexosylceramide (HexCer), dehydration ions ([M − H₂O + H] ^+^), when observed, were considered characteristic ion forms of sphingolipids under positive electrospray ionization conditions and were include in the MRM transition settings. For lysophosphatidylcholine (LPC) species, annotations were assigned according to molecular species and fatty acyl chain composition. Definitive sn-1/sn-2 positional assignment was not performed unless confirmed by authentic standards. For glyceroglycolipids such as monogalactosyldiacylglycerol (MGDG), identification was performed based on class-specific diagnostic product ions, characteristic MRM transitions, and RT information. These parameters were integrated to support the reliable identification of glycolipid species. Only lipid species meeting the predefined identification criteria were retained for subsequent quantitative analysis. For selected lipid species, annotations were further confirmed using commercially available lipid standards where available. Quantification was performed based on the peak areas of the corresponding MRM transitions on the AB Sciex QTRAP 6500 LC-MS/MS platform. Raw data were processed using Analyst 1.6.3 software, while MultiQuant software was used for peak extraction, integration, alignment, and RT correction. Lipid abundance was normalized based on internal standards.

### 2.6 Volatile compounds analysis

#### 2.6.1 Extraction of volatile compounds by headspace solid phase microextraction (HS-SPME)

These conditions were optimized in previous studies (Wu, Zhan, Tang, Li, & Duan, 2022). Briefly, after thorough homogenization, accurately weighed samples (0.2 g) were transferred into 20-mL headspace vials (Agilent Technologies, Santa Clara, CA, USA). Subsequently, 0.2 g of NaCl and 20 μL of 3-hexanone (IS-04, 10 μg/mL) internal standard solution were added. The samples were incubated at 60 °C under constant shaking for 5 min to reach equilibrium. Following equilibration, the volatiles were extracted at 250 °C for 5 min using an SPME-Arrow fiber (DVB/CWR/PDMS, 120 μm; CTC Analytics AG, Zwingen, Switzerland) and subsequently desorbed in the gas chromatography (GC) injector at 250 °C for 5 min.

#### 2.6.2 GC–MS analysis

The method described by Chen et al. was adopted with slight modifications (Chen et al., 2023). The analysis of volatiles compounds was performed using an Agilent 8890-7000D GC–MS system (Agilent Technologies, Santa Clara, CA, USA) equipped with a DB-5MS capillary column (30 m × 0.25 mm, 0.25 μm film thickness; Agilent Technologies, Santa Clara, CA, USA). The analytical conditions were set as follows: high-purity helium (≥ 99.999%) was used as the carrier gas at a constant flow rate of 1.2 mL/min. The injection port temperature was maintained at 250 °C, and the solvent delay was set to 3.5 min. The column oven temperature program was initiated at 40 °C for 3.5 min, increased to 100 °C at a rate of 10 °C/min, further raised to 180 °C at 7 °C/min, and finally ramped to 280 °C at 25 °C/min, where it was held for 5 min. The mass spectrometer was operated in selected ion monitoring (SIM) mode, monitoring the qualitative and quantitative ions precisely in accordance with the GB 23200.8-2016 standard.

#### 2.6.3 Identification and relative quantification of volatile compounds

Volatile compounds in mutton were identified by comparing their mass spectral fragmentation patterns, RT, and selected qualitative and quantitative ions with those documented in an in-house database. The mass spectrometer was operated in SIM mode to ensure focused and precise data acquisition. The relative concentrations of the volatile compounds were calculated using the internal standard method; specifically, the peak area of each target compound in the total ion chromatogram was normalized to that of the internal standard (IS-04). To monitor system stability and ensure data reliability, QC samples, prepared by pooling equal aliquots (0.2 g) of all test samples, were interspersed throughout the analytical sequence.

The relative odor activity value (ROAV) is a method for determining key flavor compounds in food, established by combing the sensory thresholds of compounds, used to clarify the contribution of each aroma compound to the overall aroma profile of the sample. Effectively evaluated the main odor compounds in different *longissimus dorsi* muscles to assess the contribution of various volatile components to the overall aroma characteristics of the sample. The higher the ROAV value of volatile flavor compounds, the greater their contribution to the sample's flavor. Components with a ROAV equal to or greater than 1 were considered significant contributors to the overall flavor (Xiao et al., 2022). The ROAVs were calculated using the following equation:

$${\mathrm{ROAV}}_{i}=\frac{C_{i}}{T_{i.}}$$

where C represents the relative concentration of a volatile component, T denotes its respective odor threshold, i signifies the detected volatile component, all units are μg/g or μg/mL.

### 2.7 Statistical analysis

Differences among groups in muscle morphological characteristics (cross-sectional area, diameter, and density), IMF content, and the concentrations of CPT1, ROS, and MDA were analyzed using one-way analysis of variance (ANOVA) in SPSS 27.0 software (SPSS, Inc., Chicago, IL, USA), followed by Tukey's honestly significant difference (HSD) test for post hoc multiple comparison. Statistical significance was set at *P*<0.05. Data visualization and graphical presentation were performed using GraphPad Prism 8.0 software (GraphPad Software Inc., La Jolla, CA, USA). For two-group analysis, differential lipids and flavor compounds were identified based on principal component analysis (PCA), orthogonal partial least squares discriminant analysis (OPLS-DA), variable importance in projection (VIP), and radar chart were employed and analyzed within R software to examine metabolite accession-specific accumulation. To evaluate the relationships between flavor compounds and key volatile compounds mantel test for correlation were employed. According to OPLS-DA analysis, the differential lipids and flavor compounds were screened by the following criteria: fold change (FC) ≥ 2 or ≤0.5 are selected; variables with VIP > 1 were considered important contributors to the model, and correlation coefficients with |r| > 0.8 and *P* < 0.05 were regarded as significant. Differences were considered statistically significant at *P* value <0.05. The aroma characteristics of different samples were comprehensively evaluated based on the identified volatile compounds and their odor properties. The ROAV was calculated to assess the contribution of individual volatile compounds to the overall aroma profile. Volatile compounds with ROAV values ≥1 were identified as key aroma-active compounds and considered major contributors to the overall aroma characteristics. According to their odor descriptors, volatile compounds were further classified into different aroma categories. The intensity of each aroma attribute was determined by integrating the contribution values of volatile compounds within the same odor category, and radar charts were generated to visualize the differences in aroma profiles among samples. Furthermore, an aroma attribute–volatile compound correlation network was constructed to explore the associations between key volatile compounds and their corresponding aroma characteristics. Each biological sample was injected once for both LC-MS/MS and GC–MS analyses, QC samples were prepared by mixing equal amounts of all samples to monitor the stability and reliability of the method described.

## 3 Results and discussions

### 3.1 Muscle histomorphology and IMF content analysis of muscle tissue in different groups

The microstructural architecture and metabolic characteristics of muscle fibers serve as fundamental determinants of meat quality, directly governing tenderness and the deposition of flavor precursors. Tenderness, a pivotal quality attribute, is modulated by multifactorial interactions involving muscle fiber diameter, connective tissue abundance, and hydration status. Reduced fiber diameter and cross-sectional area are positively correlated with enhanced meat tenderness (Huo et al., 2022). IMF is recognized as a key determinant of meat quality. Moderate increases in IMF have been associated with improved tenderness, juiciness, and overall eating quality (Wood et al., 2008). In this study, histological analysis was performed using the Case Viewer 2.3 image analysis system at 100× magnification. As illustrated in Fig. 1A-1C, the fiber diameter and cross-sectional area of the *Longissimus dorsi* muscle were significantly larger in the CK group compared to the T1 and T2 groups (*P* < 0.05). Conversely, fiber density remained comparable across all three groups (*P* > 0.05). As shown in Fig. 2A, both the T1 and T2 groups significantly increased the IMF content compared with the CK group (*P* < 0.05), no significant difference was observed between the T1 and T2 groups (*P* > 0.05). Collectively, these findings suggest that dietary SA supplementation may contribute to improved meat tenderness-related characteristics in Hu sheep.

**Fig. 1:**
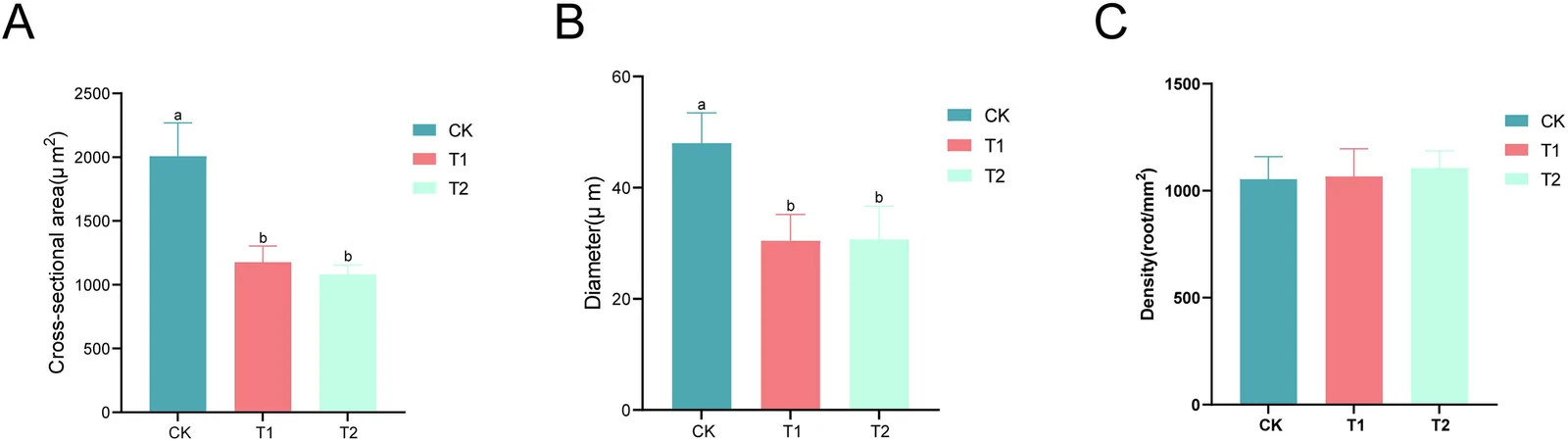
Morphological indicators of different muscle tissue groups. (A) Cross-sectional area. (B) Diameter. (C) Density. Values was presented as mean ± SD (*n* = 15 per group). Statistical followed by Tukey's HSD post hoc test. Different lowercase letters indicate significant differences among groups (*P*<0.05).

**Fig. 2:**
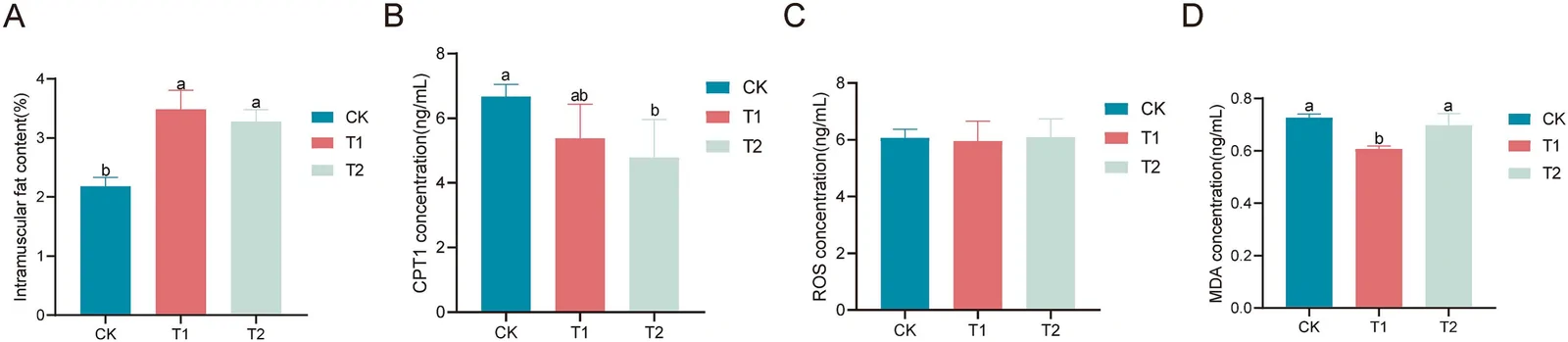
IMF content, CPT1 content, and oxidative stress marker levels in different groups. (A) IMF content. (B) CPT1 content. (C) ROS level. (D) MDA content. Values was presented as mean ± SD (*n* = 6 per group). Statistical followed by Tukey's HSD post hoc test. Different lowercase letters indicate significant differences among groups (*P*<0.05).

It is well established that the morphometric characteristics of myofibers, such as cross-sectional area and diameter, are intrinsically coupled with fiber type composition (Lee, Joo, & Ryu, 2010). Previous evidence demonstrates that dietary interventions, such as resveratrol supplementation, can remodel meat quality by modulating myofiber type transformation (Zhang et al., 2015). Specifically, a reduction in myofiber diameter is widely regarded as a positive indicator of improved meat tenderness. In the present study, we observed that the myofiber cross-sectional area in the SA substitution groups was significantly lower than that in the CK group (*P* < 0.05). This suggests that SA feeding promotes a refinement of muscle microstructure, which may be associated with improved meat texture and eating quality. Although the precise shift in fiber type composition warrants further verification via immunohistochemistry, such a reduction in myofiber dimensions is typically accompanied by adaptive changes in metabolic phenotype. This microstructural refinement may foster a metabolic microenvironment that is more conducive to IMF deposition. In this study, the IMF content was significantly higher in the SA substitution groups than in the CK group (*P* < 0.05), further supporting the potential of SA substitution to improve meat quality by promoting IMF deposition. Given that IMF serves not only as a critical reservoir of precursors for volatile flavor compounds but also as a pivotal determinant of sensory quality, these morphological and IMF alterations provide a structural basis for interpreting the subsequent lipidomic differences. Collectively, SA feeding may optimize the metabolic equilibrium between protein accumulation and lipid storage, thereby enhancing the bioavailability of flavor precursors and ultimately improving the juiciness and flavor profile of the meat (Wu, Qi, Li, Zhang, & Ma, 2026).

### 3.2 Changes in CPT1, and oxidative stress-related indicators in different groups

CPT1 catalyzes the formation of acylcarnitine from long-chain and carnitine, thereby facilitating their transport into mitochondria for β-oxidation, and serves as a key rate-limiting enzyme in fatty acid oxidation (Knottnerus et al., 2018). ROS and MDA are widely used biomarkers of oxidative stress and lipid oxidative damage. ROS reflects intracellular reactive oxygen species levels, whereas MDA is commonly used as an indicator of lipid peroxidation and oxidative stability in meat products (Ayala, Muñoz, & Argüelles, 2014; Estévez, 2015). As shown in Fig. 2B-2D, the SA substitution groups decreased CPT1 content compared with the CK group. The CPT1 content in the CK group was significantly higher than that in the T2 group (*P* < 0.05), whereas no significant differences were observed between the T1 group and the CK or T2 groups (*P* > 0.05). In addition, no significant differences in ROS levels were detected among the three groups (*P* > 0.05). Regarding MDA content, both the CK and T2 groups exhibited significantly higher levels than the T1 group (*P* < 0.05), while no significant difference was observed between the CK and T2 groups (*P* > 0.05).

Reduced CPT1 expression or activity may impair fatty acid oxidation capacity (Schlaepfer & Joshi, 2020). In this present study, CPT1 content was lower in the SA substitution groups than in the CK group, suggesting that mitochondrial β-oxidation may have been suppressed, thereby contributing to enhanced IMF deposition. Previous studies have shown that excessive mitochondrial fatty acid oxidation increases electron transport chain activity, promotes ROS generation, and subsequently induces lipid peroxidation and MDA accumulation (Schönfeld & Wojtczak, 2007). However, ROS levels remained unchanged among the groups, whereas MDA content significantly reduced in the T1 group but showed no significant difference between the CK and T2 groups. These finding suggest that SA-associated IMF deposition was not accompanied by apparent oxidative stress or enhanced lipid peroxidation.

### 3.3 Lipidomic analysis

#### 3.3.1 Changes in lipid profiles in different groups

Lipids are pivotal determinants of meat quality, dictating not only texture and juiciness but also serving as the fundamental source of volatile compounds derived from degradation during processing and maturation, which impart the characteristic flavor profile to meat products. A total of 1098 lipid compounds were identified in the three groups of samples. Table S2 shows all lipid compounds in all samples. As shown in Fig. 3A, all lipids were classified into six major classes: 526 (47.94%) GPs, 270 (24.59%) GLs, 205 (18.67%) SPs, 81(7.38%) FAs, 13 (1.18%) STs, and 3 (0.27%) PRs. GPs and GLs exhibited the highest contents, aligning with findings of (Liu et al., 2025). Among all lipids, the six major classes showed significant difference (*P* < 0.05, Fig. 3B). Specifically, the FAs content was significantly higher in the T1 group compared to the T2 group (*P* < 0.05). In contrast, the T1 group exhibited lower levels of multiple lipid classes; notably, GLs contents in the T1 group were significantly lower than those in the CK (*P* < 0.05) and T2 (*P* < 0.01) groups. Similarly, SPs levels were elevated in both the CK and T2 groups relative to T1 (*P* < 0.05). Furthermore, the CK group displayed a higher abundance of GPs (*P* < 0.01) and STs (*P* < 0.05) compared to the T1 group. No significant differences were observed in PRs content among the groups (*P* > 0.05). These lipids were further classified into 45 subclasses (Fig. 3C), including 232 (21.13%) TG, 79 (7.19%) PE-P, 76 (6.92%) PE, 69 (6.28%) PE-O, 61 (5.56%) Cer-NS, 59 (5.37%) CAR, 41 (3.73%) PG, 36 (3.28%) LPC, 36 (3.28%) PC, 35 (3.19%) SM, 34 (3.1%) PI, 30 (2.73%) PA, 30 (2.73%) PS, 25 (2.28%) Cer-NP, 21 (1.91%) Cer-AP, 21 (1.91%) DG, 21 (1.91%) Hex Cer-NS, 20 (1.82%) LPE, 20 (1.82%) PC-O,19 (1.73%) FFA, 12 (1.09%) Hex Cer-AP, 11 (1%) LPG, 10 (0.91%) LNAPE, 9 (0.82%) Cer-AS, 8 (0.73%) BA, 8 (0.73%) LPI, 7 (0.64%) LPC-O, 7 (0.64%) LPS, 7(0.64%) MGDG, 5 (0.46%) Cer-NDS, 5 (0.46%) CerP, 5 (0.46%) DG-O, 5 (0.46%) LPE-P, 4 (0.36%) CE, 4 (0.36%) Hex2Cer, 4 (0.36%) LPA, 4 (0.36%) SPH, 3 (0.27%) CoQ, 3 (0.27%) Eicosanoid, 3 (0.27%) PMeOH, 3 (0.27%) TG-O, 2 (0.18%) MG, 2 (0.18%) SHexCer, 1 (0.09%) Cer-ADS, 1 (0.09%) Cholesterol. Notably, TG, PE-P, and PE were major lipid subclasses of lipid composition in lamb. (Li et al., 2022) studies on the muscle fat of Hu sheep have found that TG affect the aroma of meat by oxidizing polyunsaturated fatty acids. These results indicate that SA substitution may alter lamb lipid composition and content, possibly leading to observed changes in lamb flavor.

**Fig. 3:**
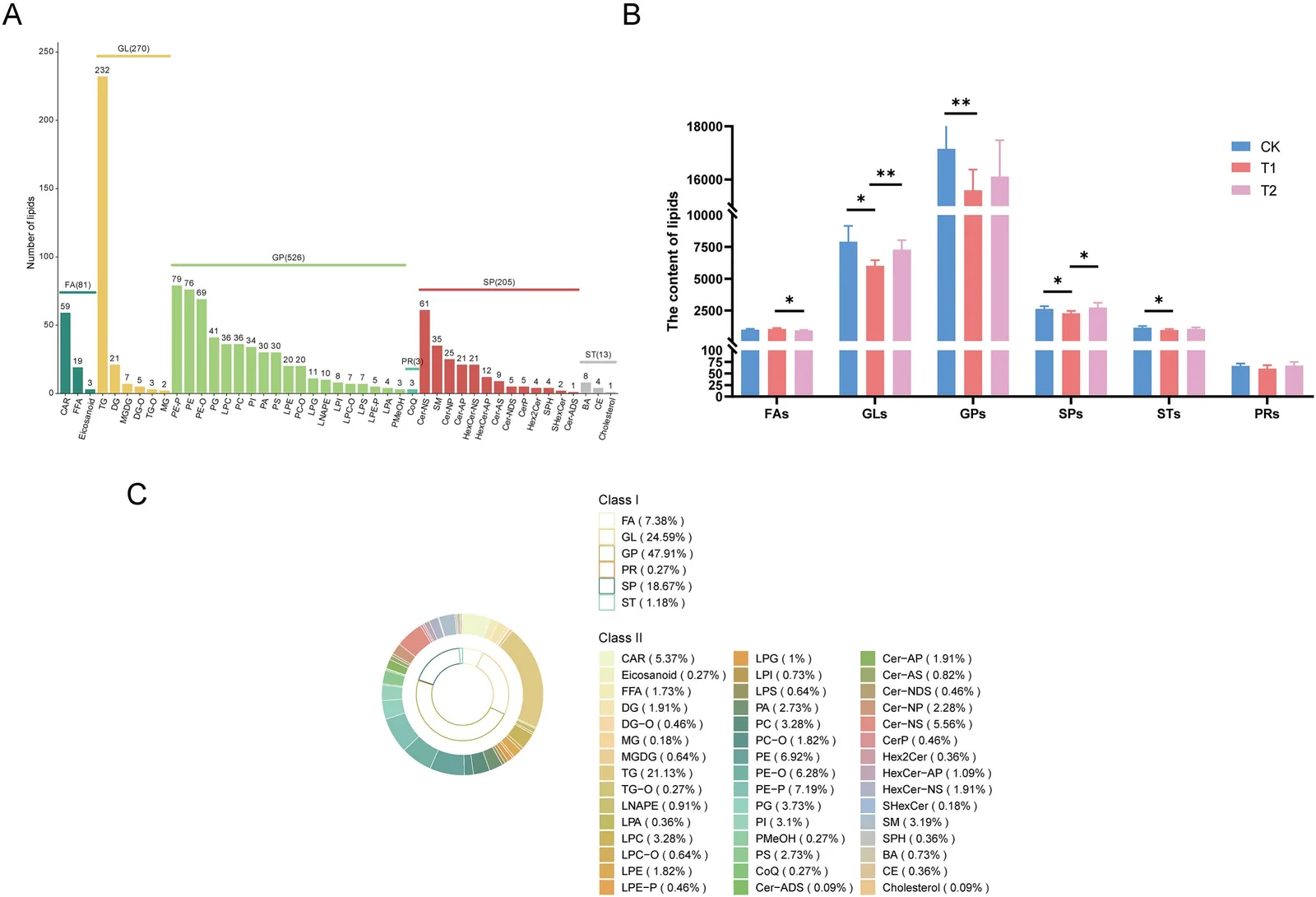
Lipid profiles in different groups. (A) Numbers of lipids species identified in 6 classes and 45 subclasses. (B) The content of differential lipid molecules and their proportions in six major classes (FAs, GLs, GPs, SPs, STs, and PRs). (C) Percentages of each lipid class and subclass.

#### 3.3.2 Screening and identification of key differential lipids

To visualize the overall distribution patterns and reveal intrinsic clustering among groups, we employed PCA and OPLS-DA. The PCA score plot showed that the first two principal components accounted for 47.84% of the total variance (Fig. 4A). The clear separation observed between groups indicates that SA substitution significantly remodels the lipid metabolic phenotype of muscle tissue. To further maximize group discrimination and screen for lipid biomarkers, OPLS-DA was performed. OPLS-DA model was a supervised multivariate statistical analysis method that combined orthogonal signal correction (OSC) and partial least squares discriminant analysis (PLA-DA) (Worley & Powers, 2016). R^2^Y is typically used to evaluate model fit, while Q^2^ is used to evaluate predictive ability. Higher values of these parameters closer to 1 indicated a more predictable or explanatory model. Typically, a Q^2^ value greater than 0.5 was considered acceptable for model predictability (Kang et al., 2022). OPLS-DA model showed that CK vs. T1 (R^2^Y = 0.996, Q^2^ = 0.936) and CK vs. T2 (R^2^Y = 0.998, Q^2^ = 0.960), demonstrating distinct metabolic segregation (Fig. 4B and D).

**Fig. 4:**
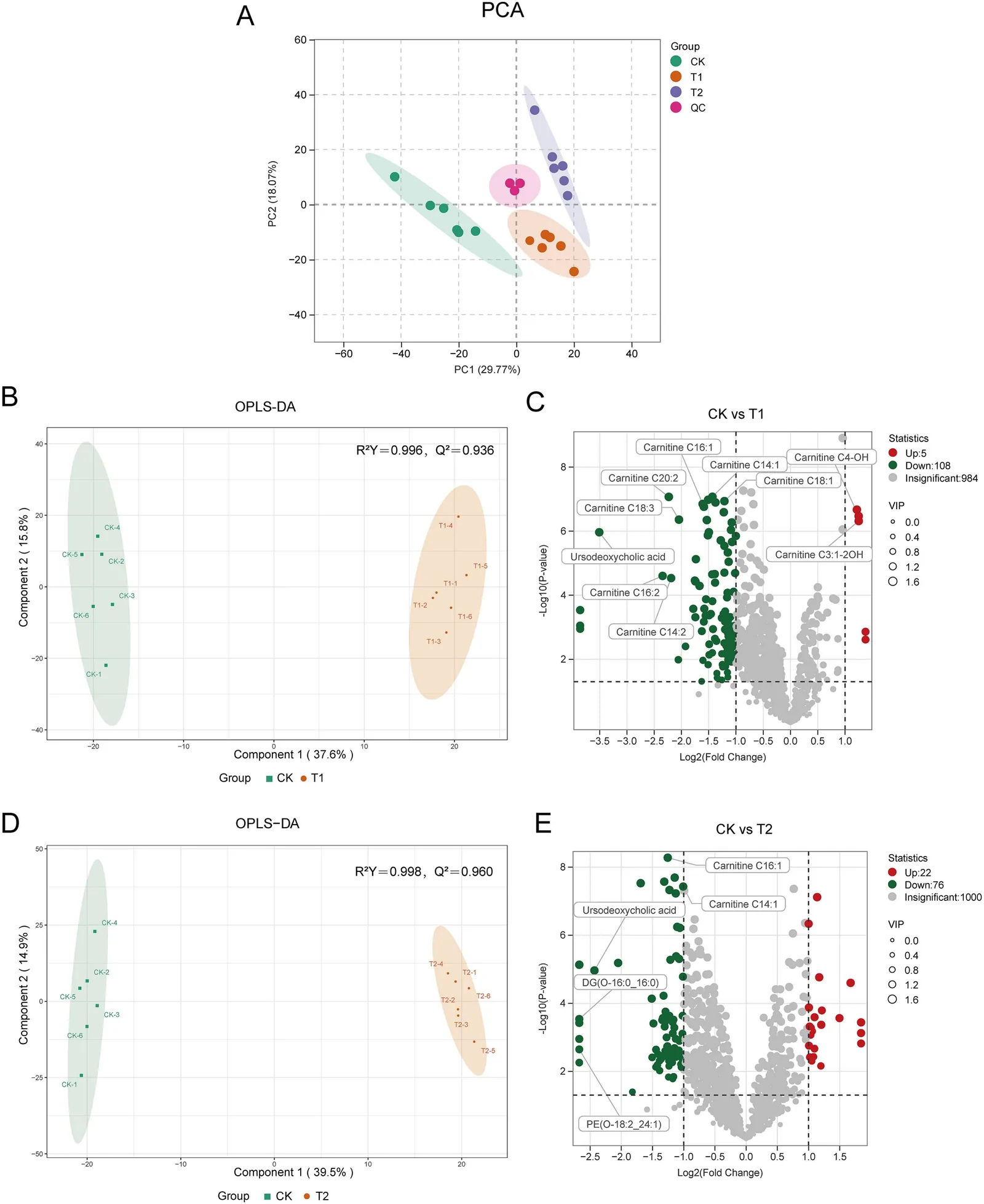
Lipidomic profiles of raw lamb from different groups by LC-MS/MS analysis. (A) PCA score plot of lipidomic profiles among the CK, T1, and T2 groups with the QC samples. (B) OPLS-DA score plot of lipidomic profiles between the CK and T1 groups. (C) Volcano plot showing differential lipid metabolites between the CK and T1 groups. Red and green dots represent significantly increased and decreased metabolites, respectively. (D) OPLS-DA score plot of lipidomic profiles between the CK and T2 groups. (E) Volcano plot showing differential lipid metabolites between the CK and T2 groups. (For interpretation of the references to color in this figure legend, the reader is referred to the web version of this article.)

Important volatile compounds were screened based on their VIP value extracted from OPLS-DA model. Based on the VIP > 1, FC ≥ 2 or ≤ 0.5, and *P* < 0.05 as selection criteria, screening for differential lipids. Volcano plots illustrate the identification of 113 and 98 differential lipids in the CK vs. T1 and CK vs. T2 comparisons, respectively (Fig. 4C and E). Table S3 and Table S4 shows these lipids were primarily categorized into glycerides, glycerophospholipids, and fatty acyls. Neutral fats and phospholipids are two types of lipids in lamb that affect taste perception; they are converted into aromatic compounds with strong odors (Chevance & Farmer, 1999). Unlike triglycerides (neutral fats), which have a lower degree of unsaturation, glycerophospholipids are rich in polyunsaturated fatty acids (PUFAs). Hydrolysis of these phospholipids releases linoleic and linolenic acids, serving as key substrates for the generation of characteristic volatile flavor compounds. Furthermore, as essential components of the cell membrane scaffold, the oxidative degradation of phospholipid acyl chains directly yields volatile aldehydes and ketones. These compounds modulate the adsorption kinetics of flavor molecules on the oral mucosa, thereby influencing the sensitivity of flavor perception (Dyall et al., 2022).

Notably, in the CK vs. T1 comparison, we observed a consistent downregulation of long-chain acylcarnitines (Carnitine C14:1, Carnitine C16:1, Carnitine C16:2, Carnitine C18:1, Carnitine C18:3, Carnitine C20:2) and Ursodeoxycholic acid alongside a significant upregulation of short-chain acylcarnitines (Carnitine C4-OH, Carnitine C3:1-2OH) (Fig. 4C). The carnitine shuttle system represents the rate-limiting step for the transport of LCFAs into the mitochondrial matrix for β-oxidation (Kerner & Hoppel, 2000). Based on the data, the suppression of long-chain acylcarnitines suggests that SA intake CPT1 activity or restricts carnitine transport kinetics, effectively attenuating mitochondrial fatty acid oxidation flux. Consequently, fatty acids spared from catabolism are metabolically redirected toward the synthesis and sequestration of triglycerides and phospholipids, explaining the prevalence of these lipid classes in the differential profile. Plants are rich sources of secondary metabolites, including terpenoids, flavonoids, and alkaloids, which have been reported to modulate mitochondrial function and related metabolic processes (Masyita et al., 2022; Shen et al., 2022). Therefore, these bioactive compounds in SA may collectively contribute to the metabolic remodeling observed in the present study. Although detailed profiling of the SA phytochemical landscape remains to be elucidated, the metabolic phenotypes observed here are consistent with the known bioactivities of such plant-derived compounds. This metabolic diversion facilitates the deposition of IMF, a key determinant of tenderness and juiciness (Natalello et al., 2023). Therefore, the reduction of long-chain acylcarnitines following SA supplementation suggests a potential shift in fatty acid utilization from mitochondrial oxidation toward lipid storage pathways, which may contribute to increased IMF accumulation and altered flavor precursor availability. The accumulation of short-chain acylcarnitines (e.g., Carnitine C4-OH) likely mirrors an increase in rumen-derived short-chain fatty acids (e.g., butyrate), which serve as compensatory energy substrates for myocytes when LCFAs oxidation is suppressed, ensuring energy homeostasis while prioritizing LCFAs deposition. In the CK vs. T2 comparison, five key lipids were significantly downregulated, including long-chain acylcarnitines, ursodeoxycholic acid, and specific ether lipids [PE(O-18:2_24:1)] (Fig. 4E). The reduction in ursodeoxycholic acid suggests that SA protein may alter enterohepatic circulation or lipid digestion efficiency. Crucially, PE(O-18:2_24:1) is a plasmalogen species, a unique class of ether phospholipids characterized by a vinyl-ether bond at the sn-1 position, which confers intrinsic antioxidant properties (Chen et al., 2025). The altered abundance of this lipid suggests potential remodeling of membrane lipid composition and plasmalogen-associated oxidative regulation following SA substitution. Yu et al. (2024) found that addition of perilla (rich in flavonoids, terpenes, polyphenols, and amino acids) to Tan sheep (*Ovis aries*) diets was found to increase the content of omega-3 PUFAs and decrease the omega-6/omega-3 ratio, while decreasing the LPCs, increasing the Cos, and improving the flavor of the lamb. Dietary polyphenols can accumulate in muscle tissue, acting as direct free radical scavengers and regulating the expression of antioxidant enzymes such as superoxide dismutase. Therefore, this protection of PUFAs from peroxidation not only prevents rancidity but also promotes the controlled generation of desirable volatile compounds, such as specific esters and lactones.

### 3.4 Volatile compounds analysis

The characteristic aroma of mutton arises from the thermal transformation of key precursors (including amino acids, reducing sugars, and nucleotides) via the Maillard chemistry, thermal decomposition, and fat oxidation (Yang et al., 2022). This research investigated the volatile flavor profiles of three groups by employing HS-SPME-GC–MS. Table S5 shows a total of 535 volatile compounds with CAS annotations were characterized in the three groups, including 151 aldehyde, ketones, esters, 117 hydrocarbons, 87 alcohol and amines, 54 heterocyclic compounds, 46 benzene and substituted derivatives, 45 terpenoids, 14 organic acid and its derivatives, 10 nitrogen compounds, 9 ethers, and 2 halogenated hydrocarbons (Fig. 5A). Based on their relative content and type, aldehyde, ketones, and esters were determined to be the primary volatile flavor compounds in the three groups. Aldehydes, characterized by low odor thresholds and high volatility, are widely recognized as primary determinants of flavor profiles (Elmore et al., 2005). During lipid oxidation, unsaturated fatty acids undergo heat- or enzyme-induced free-radical chain reactions to yield hydroperoxides, which subsequently decompose into chain-length-specific aldehydes, such as hexanal (grassy), octanal (fatty), and nonanal (citrus-like) (Zhuang et al., 2022). Ketones, synthesized synergistically via fatty acid β-oxidation and the Maillard reaction, serve as the primary sources of buttery and fresh-sweet fruity notes, significantly enhancing the olfactory complexity of lamb (Bleicher et al., 2022). Furthermore, β-ionone category (e.g., β-ionone, trans-β-ionone), derived from the oxidative cleavage of carotenoids, act as potent odorants with extremely low detection thresholds, defining the premium flavor profile of meat (Gallon et al., 2013). Esters, ubiquitous in fresh fruits and flowers, contribute characteristic creamy and floral-fruity nuances (Gao et al., 2017). Previous studies have documented age-dependent ester profiles in Jingyuan lamb, identifying butyl butyrate in 2-month-old lambs and ethyl acetate and amyl acetate in 6-month-old lambs, confirming their role in fruity aroma formation (Wang et al., 2021). In the present study, the T1 group exhibited a significantly higher relative abundance of 3,6-Nonadien-1-ol, (E, Z)- (cucumber/fresh green) and specific lactones (e.g., 2(5H)-Furanone, 5-(1-methylethyl)-, fruity). The observed changes may reflect a metabolic shift in which reduced mitochondrial fatty acid oxidation is accompanied by increased allocation of fatty acids toward cytoplasmic esterification pathways. Consistent with (Vasta & Priolo, 2006), dietary components may also facilitate the direct transfer of specific volatiles into adipose tissue. Collectively, these findings suggest that SA feeding may reshape mitochondrial fatty acid utilization and lipid allocation, thereby flavoring lipid accumulation and contributing to the altered abundance of aroma-associated aldehydes, ketones, and esters. Although hydrocarbons are also generated via the oxidative degradation of lipids (particularly PUFAs), saturated hydrocarbons typically possess high sensory thresholds and thus make a limited contribution to the overall volatile profile. Ultimately, this specific metabolic remodeling underpins the unique flavor phenotype observed in SA-fed lamb.

**Fig. 5:**
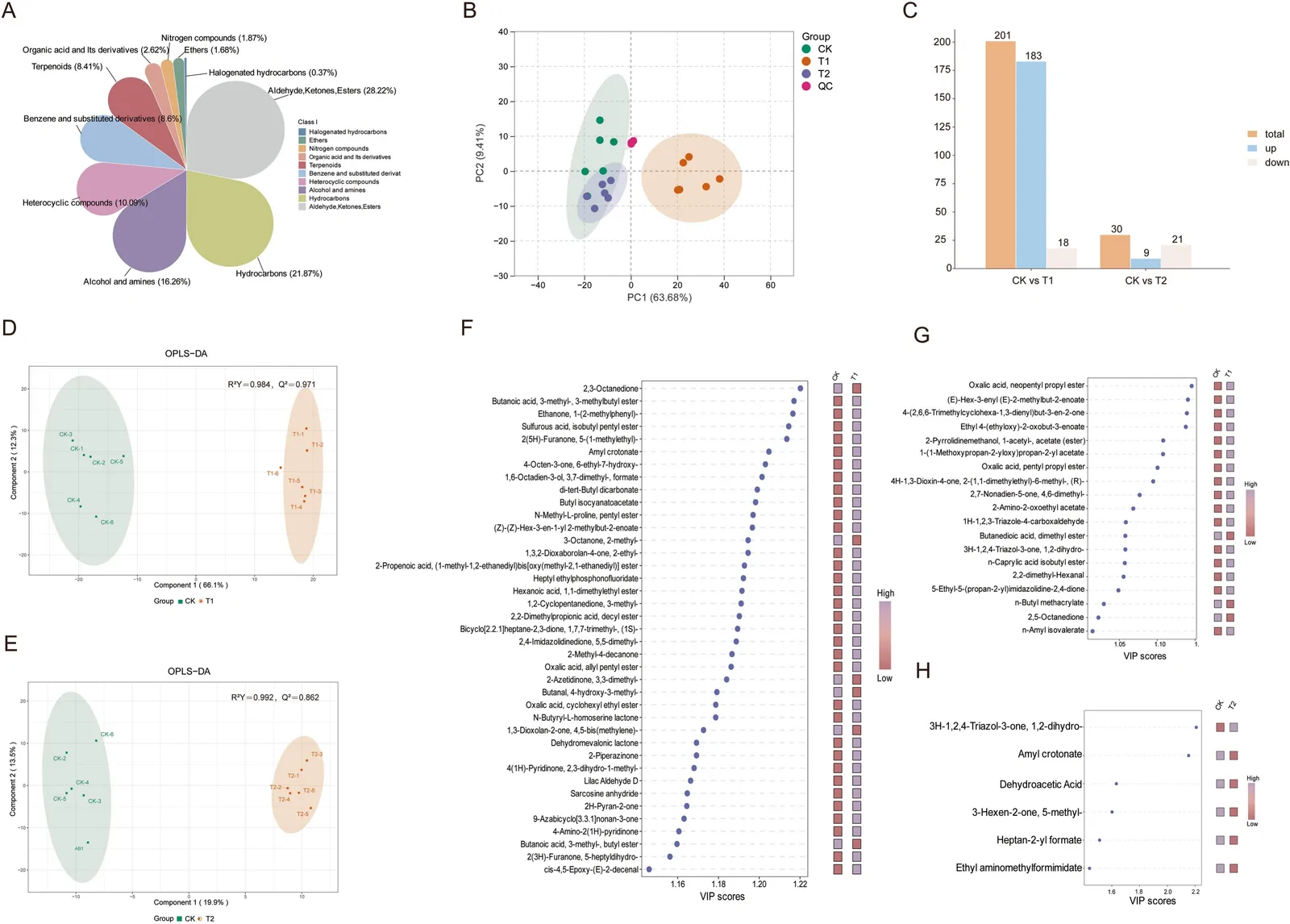
Volatile compounds profiles of raw lamb from different groups by GC–MS analysis. (A) Composition of identified VOC classes. (B) PCA score plot of volatile compounds among the CK, T1, and T2 groups and QC samples. (C) Numbers of increased and decreased volatile compounds identified in the CK vs. T1 and the CK vs. T2 comparisons. (*P* < 0.05). (D) OPLS-DA score plot of volatile compounds between the CK and T1 groups. (E) OPLS-DA score plot of volatile compounds between the CK and T2 groups. (F-G) VIP score of differential volatile compounds identified in the CK vs. T1. (H) VIP scores of differential volatile compounds identified in the CK vs. T2. Differential volatile compounds were screened based on VIP > 1 and *P* < 0.05.

To further evaluate the distribution patterns and group separation of volatile flavor compounds among the different groups, PCA was performed (Fig. 5B). The CK and T1 groups exhibited marked spatial divergence, reflecting substantial heterogeneity in their metabolic phenotypes. In contrast, the CK and T2 groups clustered tightly, indicating a high degree of similarity in their volatile signatures. The OPLS-DA model showed a clear separation between the CK and T1 groups (R^2^Y = 0.984, Q^2^ = 0.971) and between the CK and T2 groups (R^2^Y = 0.992, Q^2^ = 0.862) (Fig. 5D and E), indicating excellent model fitness and predictive ability. Collectively, these findings underscore that altering the roughage source exerts a far more profound efficacy in reshaping the volatile metabolome than altering the protein source. To isolate specific discriminants, key volatile compounds were screened based on VIP scores derived from the OPLS-DA model, key volatile compounds were screened using VIP > 1, FC ≥ 2 or ≤ 0.5, and *P* < 0.05 as screening criteria. Table S6 and S7 shows this analysis identified 201 and 30 differential volatile compounds in the CK vs. T1 and CK vs. T2 comparisons, respectively (Fig. 5C), further corroborating the extensive metabolic reprogramming induced by replacing alfalfa with SA. Specific molecular shifts were highlighted in Fig. 5F-5H. Within the differential aldehydes, ketones, and esters identified between the CK and T1 groups (Fig. 5F and G), 2,3-octanedione and Butanedioic acid, dimethyl ester were significantly downregulated in the T1 group (*P* < 0.05). Conversely, desirable flavor compounds, including 2(3H)-Furanone, 5-heptyldihydro-, (*E*)-Hex-3-enyl (E)-2-methylbut-2-enoate, and Butanoic acid, 3-methyl-, 3-methylbutyl ester were significantly upregulated in the T1 group (*P* < 0.05). Collectively, these findings suggest that SA substitution may remodel the volatile flavor profile of lamb by altering lipid metabolism, characterized by reduced accumulation of lipid oxidation-associated compounds and increased abundance of desirable aroma-active esters and lactones. The observed flavor changes are consistent with a potential shift in fatty acid utilization from mitochondrial oxidation toward lipid storage pathways. Within the differential aldehydes, ketones, and esters identified between the CK and T2 groups (Fig. 5H), 3H-1,2,4-Triazol-3-one,1,2-dihydro- was significantly downregulated in the T2 group (*P* < 0.05). Amyl crotonate, Dehydroacetic Acid, 3-Hexen-2-one, 5-methyl-, Heptan-2-yl formate, and Ethyl aminomethylformimidate were significantly upregulated in the T2 group (*P* < 0.05). In contrast to the T1 group, the T2 group was associated with relatively limited alterations in the volatile flavor profile, with changes mainly occurring in specific ester, ketone, and nitrogen-containing compounds. These findings suggest that the dietary replacement strategy influences the extent and pattern of SA-mediated flavor modulation, with protein source substitution exerting a more selective effect on volatile compound formation.

### 3.5 Determination of key flavor compounds

Odor activity value (OAV) is widely recognized as a robust metric for flavor assessment, bridging the gap between chemical composition and sensory perception (Yu et al., 2024). Given the matrix complexity inherent to volatile-rich systems, the ROAV serves as a pragmatic proxy for approximating absolute concentrations (Sun et al., 2023). In this study, we characterized the ROAV profiles across the three experimental groups, identifying 43 volatile compounds with ROAV ≥1 (Table S8). Specifically, the CK, T1, and T2 groups contained 40, 39, and 38 key odorants. In the CK group, lipid oxidation products such as nonanal and dihydro-2-methyl-3(2H)-furanone exhibited high ROAV. These aldehydes and ketones, primarily derived from the oxidative degradation of oleic (C18:1) and linoleic (C18:2) acids, are the principal contributors to the rancid and oily notes typical of mutton fat (Van Ba, Amna, & Hwang, 2013). Notably, dihydro-2-methyl-3(2H)-furanone and 2-methoxy-3-(2-methylpropyl)-pyrazine demonstrated the highest odor contributions and lowest detection thresholds, establishing green, sweet, and fatty notes as dominant sensory attributes. Intriguingly, while T1 and T2 groups shared 2-methoxy-3-(2-methylpropyl)-pyrazine as a dominant compound, the contribution of dihydro-2-methyl-3(2H)-furanone was significantly attenuated in the T1 group. This metabolic shift engendered a distinctively sweeter aroma profile in the T1 group, whereas the T2 group retained a more pronounced lipid-associated fragrance. ROAV analysis thus reveals that SA feeding drives a fundamental transition in lamb flavor from an oil-dominant to a fresh, distinct profile.

Although individual key odorants possess distinct attributes, the final flavor phenotype is governed by their complex interactions rather than their isolated presence. To comprehensively delineate the flavor remodeling effect of SA, 11 representative aroma compounds with ROAV ≥1 were selected, including β-Ionone; Pantolactone; 3(2H)-Furanone, dihydro-2-methyl-; Pyrazine, 2-methoxy-3-(2-methylpropyl)-; 1-Octen-3-one; n-Amyl isovalerate; Nonanal; 3,6-Nonadien-1-ol, (E, Z)-; Butanedioic acid, dimethyl ester; 2-Octen-1-ol; 2(3H)-Furanone, 5-heptyldihydro-. Taxonomically, aldehydes contribute fried, fatty, and fresh notes; esters dominate the characteristic lamb profile with green, fruity, and floral attributes; while ketones and heterocyclic compounds impart floral and roasted nuances. Crucially, terpenoids (e.g., β-ionone), which are synthesized exclusively in plants, contribute herbal, woody, and green accents (Mewalal et al., 2017). These bioactive molecules are transferred from the diet to animal tissues, modifying the textural quality of the flavor despite their low abundance. Consistent with (Rivaroli et al., 2016), who found that essential oils improve lipid stability, the presence of terpenoids here suggests a similar protective mechanism. To further elucidate the sensory implications of the altered volatile profiles, sensory attribute mapping was performed based on the key odor-active compounds identified by ROAV analysis (Fig. 6A and B). Compared with the CK group, the T1 group exhibited enhanced green, sweet, and fruity aroma attributes, accompanied by reduced fatty notes, indicating a shift toward a fresher and more desirable flavor profile associated with SA substitution for alfalfa. In contrast, the T2 group displayed a distinct but more limited sensory alteration, characterized by increased woody and citrus-like notes, suggesting that SA substitution for protein supplements exerted a more selective influence on aroma characteristics. An aroma network was constructed by integrating key odorants with their corresponding sensory descriptors to visualize the relationships between volatile compounds and perceived aroma attributes (Fig. 6C). The network analysis revealed that Butanoic acid, 3-methyl-, 3-methylbutyl ester, Butanedioic acid, dimethyl ester, butyl ester, and 5-heptyldihydro-2(3H)-furanone were primarily associated with fruity and sweet aroma attributes. In contrast, 2,3-octanedione and (*E*)-2-octen-1-ol showed strong associations with fatty aroma characteristics, suggesting their potential contribution to lipid-derived aroma formation. Furthermore, terpene-related and plant-associated volatile compounds, including β-ionone, (E)-.beta.-Famesene, Isborneol, and related compounds, were closely associated with woody, herbal, and fresh aroma attributes, suggesting that dietary-derived bioactive compounds may contribute to the formation of distinct aroma profiles. Collectively, these findings indicate that SA not only alters lipid metabolism but also contributes to distinct aroma modulation patterns depending on the dietary substitution strategy.

**Fig. 6:**
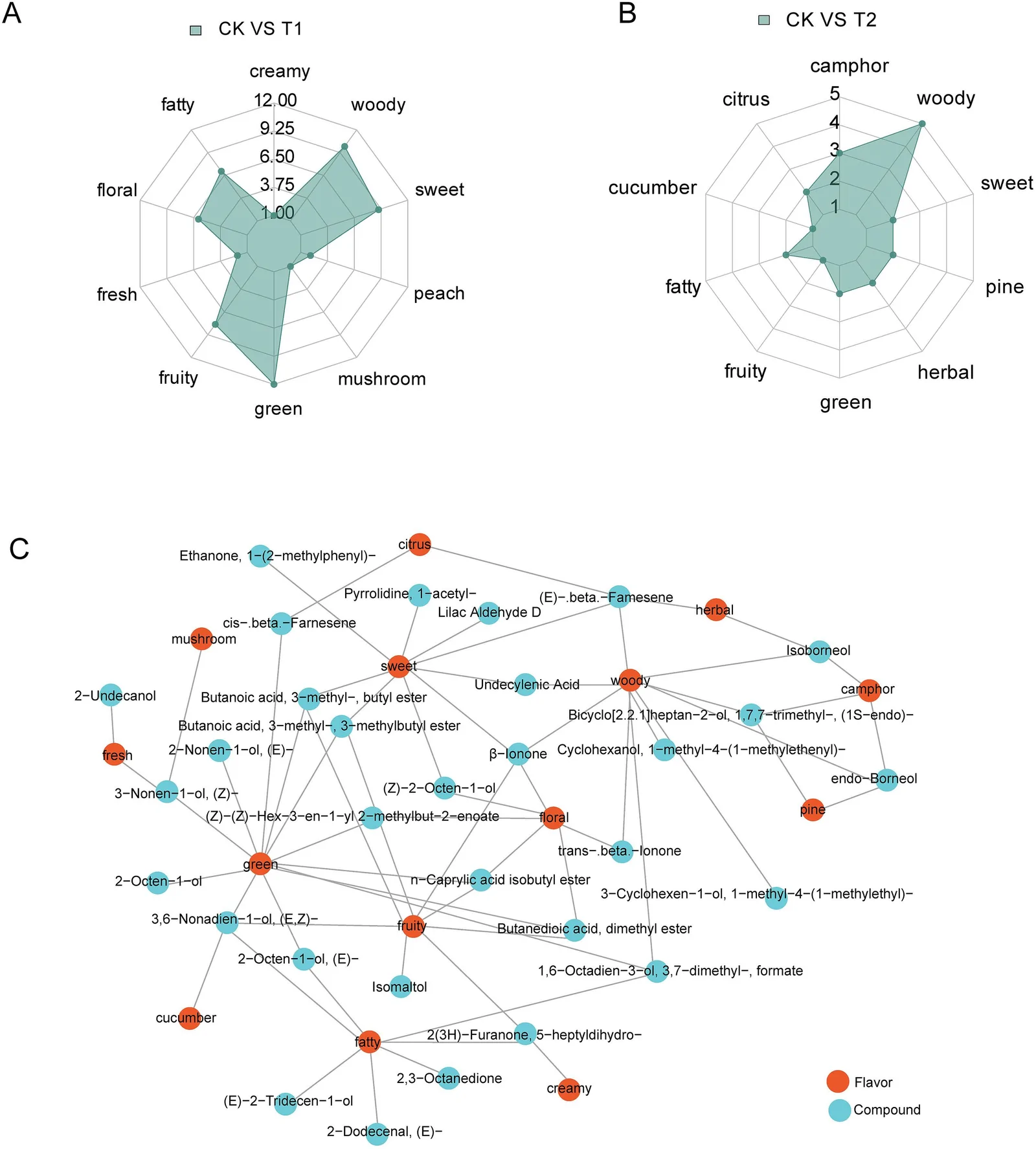
The sensory odors characterization among different group. (A). CK vs. T1. (B). CK vs. T2. (C). Correlation network between the aroma compounds and sensory odors description.

### 3.6 Correlation analysis between aromas and lipids

Meat aroma genesis is underpinned by complex chemical transformations of specific flavor precursors. Given our prior observation of concurrent shifts in lipidomic and volatile profiles, we hypothesized a direct regulatory link between the carnitine shuttle system and flavor development. To test this, we performed a Mantel test-based correlation analysis between differential lipid species and key volatile compounds (Fig. 7A and B). The resulting network visualization, where edge width and color denote Mantel's correlation coefficient (r) and statistical significance (p) respectively, revealed extensive associations between the lipid and volatile matrices. Specifically, long-chain acylcarnitines (e.g., Carnitine C16:1, Carnitine C18:1, Carnitine C20:2) exhibited robust positive correlations with lipid oxidation-derived volatiles, such as nonanal and 2,3-octanedione-(E). Concurrently, a distinct cluster of volatile compounds, including 2-octen-1-ol, β-ionone, isoborneol, and trans-β-ionone, which introduced fresh, floral, and cooling sensory nuances. These notes synergistically enriched the flavor hierarchy while attenuating undesirable gamey odors. Collectively, these data substantiate that the modulation of lipid abundance, driven by SA substitution, serves as the mechanistic driver for the observed remodeling of the meat aroma profile.

**Fig. 7:**
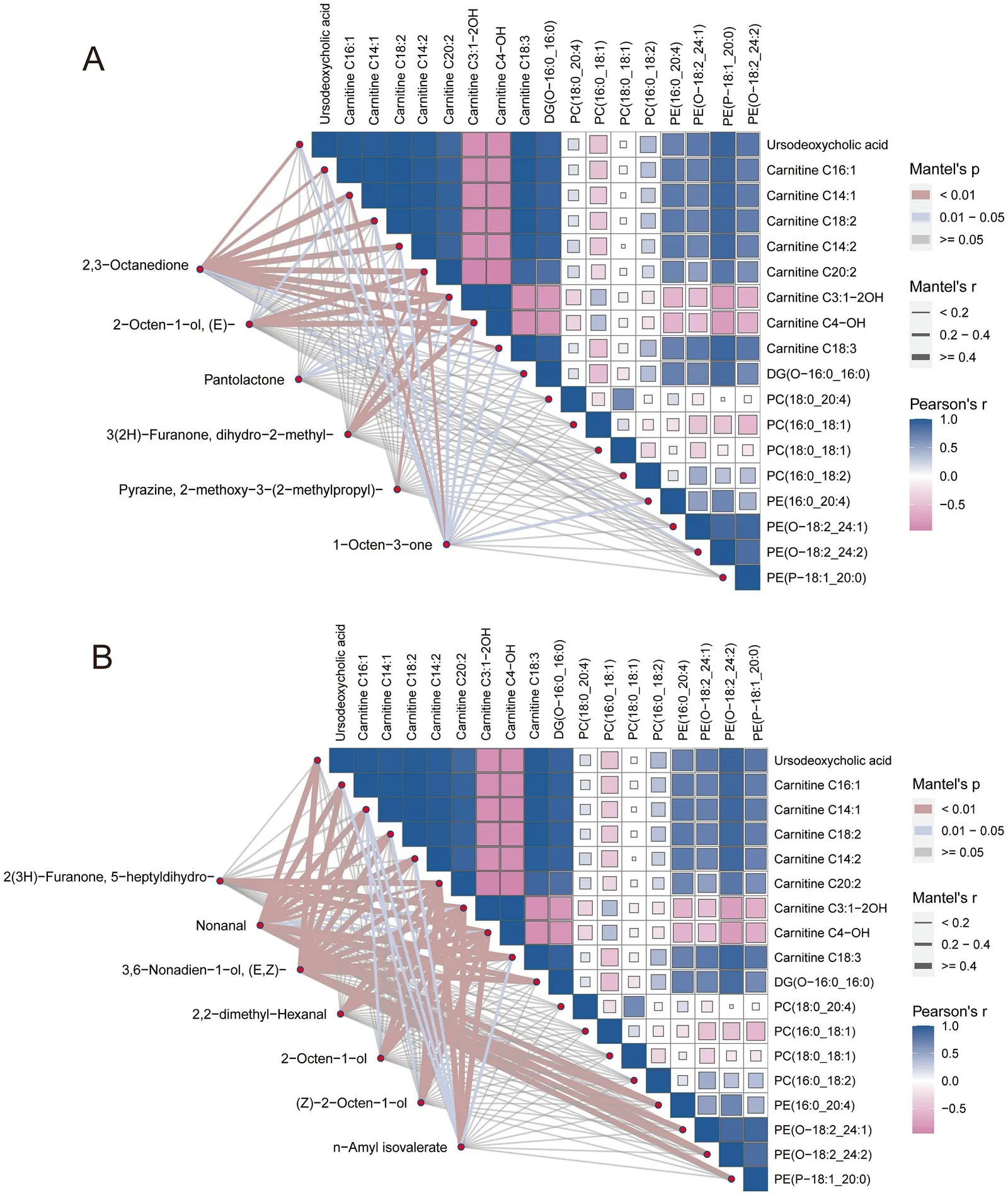
(A-B) Mantel test and Pearson correlation analysis between representative aromas and lipids metabolites. The heatmap shows Pearson correlation coefficients (r) between lipid metabolites, with blue and pink representing positive and negative correlations, respectively. The connecting lines represent the Mantel test results between aroma compounds and lipid metabolites; line color indicates Mantel significance (*p* < 0.01, 0.01 ≤ *p* < 0.05, and *p* ≥ 0.05), and line width represents the Mantel correlation coefficient (*r* < 0.2, 0.2 ≤ *r* < 0.4, and *r* ≥ 0.4). Mantel tests were performed with six biological replicates in each group (*n* = 6). (For interpretation of the references to color in this figure legend, the reader is referred to the web version of this article.)

## 4 Conclusions

This study investigated the effects of SA substitution on lipid composition and volatile flavor profiles in Hu sheep muscle and explored the potential role of lipid metabolism in SA-mediated flavor regulation. Compared with the CK group, SA substitution increased IMF content. Lipidomic analysis revealed that long-chain acylcarnitines, including Carnitine C16:1, C18:1, and C20:2, were significantly decreased in SA-substituted groups, suggesting altered fatty acid partitioning between mitochondrial oxidation and lipid storage pathways, thereby influencing the availability of flavor precursors. ROAV analysis (ROAV ≥1) identified 43 key odor-active compounds, with the T1 group exhibiting enhanced green, sweet, and fruity aroma attributes, whereas the T2 group showed a more pronounced woody aroma profile compared with the CK group. These findings indicate that SA supplementation reshaped the lamb aroma profile toward fresher and more distinctive characteristics. Correlation analysis further revealed significant associations between long-chain acylcarnitines and lipid oxidation-related volatile compounds, including nonanal and 2,3-octanedione, highlighting the potential contribution of lipid metabolic remodeling to flavor formation. Collectively, these findings provide new insights into SA-mediated regulation of lamb flavor formation and support its potential application as a nutritional strategy for improving mutton flavor quality. Future studies incorporating sensory evaluation, key enzyme activity measurements, and dynamic sampling across slaughter stages are required to further validate the underlying mechanisms.

## CRediT authorship contribution statement

**Yajing Nie:** Writing – review & editing, Writing – original draft, Validation, Methodology, Data curation. **Liming Zhang:** Resources. **Chengxing Han:** Resources. **Xujie Li:** Resources. **Yi Wu:** Software. **Chunhui Wang:** Methodology. **Youji Ma:** Writing – review & editing, Visualization, Project administration, Funding acquisition, Formal analysis, Data curation, Conceptualization.

## Ethical statement

The animals used in this study were approved by the Animal Committee of Gansu Agricultural University (GSAU-Eth-ASF2022-008) and performed the NIH (National Research Council) Guide for the Care and Use of Laboratory Animals.

## Declaration of competing interest

The authors declare that they have no known competing financial interests or personal relationships that could have appeared to influence the work reported in this paper.

## Data Availability

Data will be made available on request.

## Appendix A Supplementary data

Supplementary Table S1 Information of lipid internal standards used for LC-MS/MS analysis

Supplementary Table S2 All lipid compounds in all samples

Supplementary Table S3 113 differential lipids in the CK vs. T1 comparisons

Supplementary Table S4 98 differential lipids in the CK vs. T2 comparisons

Supplementary Table S5 A total of 535 volatile compounds with CAS annotations were characterized in the three groups

Supplementary Table S6 201 differential volatile compounds in the CK vs. T1 comparisons

Supplementary Table S7 30 differential volatile compounds in the CK vs. T2 comparisons

Supplementary Table S8 43 volatile compounds with ROAV≥1
